# Surgical Treatment and Outcomes of Colorectal Cancer Patients During the COVID-19 Pandemic: A National Population-based Study in England

**DOI:** 10.1097/AS9.0000000000000071

**Published:** 2021-06-10

**Authors:** Angela Kuryba, Jemma M. Boyle, Helen A. Blake, Ajay Aggarwal, Jan van der Meulen, Michael Braun, Kate Walker, Nicola S. Fearnhead

**Affiliations:** From the * Clinical Effectiveness Unit, Royal College of Surgeons of England, London, United Kingdom; † Department of Health Services Research and Policy, London School of Hygiene and Tropical Medicine, London, United Kingdom; ‡ Department of Clinical Oncology, Guy’s & St Thomas’ NHS Trust, London, United Kingdom; § Department of Medical Oncology, The Christie NHS Foundation Trust, Manchester, United Kingdom; ‖ Department of Surgery, Cambridge University Hospitals NHS Foundation Trust, Cambridge, United Kingdom.

**Keywords:** COVID-19, colorectal cancer, surgical mortality

## Abstract

To compare the management and outcomes of colorectal cancer (CRC) patients during the first 2 months of the COVID-19 pandemic with the preceding 6 months.

**Background:**

The pandemic has affected the diagnosis and treatment of CRC patients worldwide. Little is known about the safety of major resection and whether creating “cold” sites (COVID-free hospitals) is effective.

**Methods:**

A national study in England used administrative hospital data for 14,930 CRC patients undergoing surgery between October 1, 2019, and May 31, 2020. Mortality of CRC resection was compared before and after March 23, 2020 (“lockdown” start).

**Results:**

The number of elective CRC procedures dropped sharply during the pandemic (from average 386 to 214 per week), whereas emergency procedures were hardly affected (from 88 to 84 per week). There was little change in characteristics of surgical patients during the pandemic. Laparoscopic surgery decreased from 62.5% to 35.9% for elective and from 17.7% to 9.7% for emergency resections. Surgical mortality increased slightly (from 0.9% to 1.2%, *P* = 0.06) after elective and markedly (from 5.6% to 8.9%, *P* = 0.003) after emergency resections. The observed increase in mortality during the first phase of the pandemic was similar in “cold” and “hot” sites (*P* > 0.5 elective and emergency procedures).

**Conclusions:**

The pandemic resulted in a 50% reduction in elective CRC procedures during the initial surge and a substantial increase in mortality after emergency resection. There was no evidence that surgery in COVID-free “cold” sites led to better outcomes in the first 2 months.

## Abstract 2

**Mini-abstract:** Elective surgery for colorectal cancer dropped by 50% at the start of the COVID-19 pandemic in England. Mortality after major resection increased from 0.9% to 1.2% for elective and from 5.6% to 8.9% for emergency procedures. There was no evidence that using COVID-free hospitals improved outcomes during the initial surge.

## INTRODUCTION

The COVID-19 pandemic has had a major impact on cancer care due to diversion of healthcare resources to treat COVID-19 patients and changes in healthcare-seeking behavior of patients with symptoms of cancer.^1, 2^ This has led to severe delays in the diagnosis and treatment of cancer patients worldwide.^3, 4^ Reported increases in the risk of surgical treatment linked to COVID-19 infection and lack of normal perioperative healthcare facilities prompted an increased use of nonoperative treatments.^5,6^

Early reports suggested that cancer patients are at greater risk of contracting COVID-19 and that they have worse COVID-19 outcomes.^7–10^ It was estimated that up to a quarter of patients with perioperative COVID-19 infection undergoing any type of surgery would die, with four-fifths of these deaths due to pulmonary complications, further contributing to a reluctance to offer elective surgery when it might reasonably be delayed to a safer time.^11, 12^

There is little evidence on the safety of colorectal cancer (CRC) surgery during the COVID-19 pandemic. A few relatively small studies have suggested that it may be possible to operate safely.^13–16^ Measures used to reduce the risks related to perioperative COVID-19 for patients undergoing planned cancer surgery include preoperative isolation and COVID-19 swab testing.

In the English National Health Service (NHS), one approach to reduce the risk of patients acquiring COVID-19 while in hospital has been the separation of hospitals into “cold” sites used for patients free of COVID-19 and “hot” sites used for all other patients.^17, 18^ Preoperative isolation and COVID-19 swab testing were used in patients having surgery in “cold” and “hot” sites to reduce the risk of community acquired infection. The effectiveness of separating CRC services in this way is uncertain. Also, it is often not feasible to redirect patients who present with CRC as an emergency to a specific site for urgent lifesaving intervention.

The aim of this study was to quantify changes in the surgical management and surgical mortality of CRC patients during the initial peak of the COVID-19 pandemic period and to assess whether the separation of hospitals into “hot” and “cold” sites was effective in reducing the COVID-19 risk in the first wave of the pandemic. Our study included a highly representative population as we studied all patients admitted for CRC surgery in the NHS, the publicly funded national healthcare system that provides more than 95% of healthcare in England, including NHS patients treated in the independent sector.^19^

## METHODS

### Data Source

The Hospital Episode Statistics (HES) database contains records of all admissions to NHS hospitals and of NHS patients treated in independent sector hospitals in England.^20^ These records include diagnostic information coded according to the International Classification of Diseases, 10th revision (ICD-10).^21^ Procedure information is coded according to the Office of Population Censuses and Surveys Classification of Surgical Operations and Procedures, 4th Revision (OPCS-4).^22^

### Study Population

All patients identified in HES as having been admitted with CRC (ICD-10: “C18,” “C19,” or “C20”) who had a surgical CRC procedure (major resection, stoma or stent, OPCS-4: “H04-H11,” “H29,” “H33,” “H40,” “H411,” “H414,” “H471,” “H479,” “X141,” “X142,” “X143,” “X148,” and “X149”) between October 1, 2019, and May 31, 2020, were included. For each patient, the earliest surgical procedure was used. Patients who underwent a stoma or stent procedure in addition to a major resection were classified as having had a major resection. When multiple resections occurred on the same date, the first listed resection was used.

### COVID-19 Pandemic Period

Patients were considered to have been treated during the initial wave of the COVID-19 pandemic if their surgical procedure was on or after March 23, 2020. This date corresponds to the start of the “lockdown” period, during which the UK government banned all nonessential travel and contact with people outside one’s home, and almost all schools and businesses were closed.^23^

### Patient Characteristics

Four-character ICD-10 codes provided the detailed tumor site, and four-character OPCS-4 codes provided the type of surgical procedure. HES records include the mode of admission (“elective” and “emergency”), and we assumed that procedures in an elective admission were elective procedures and those in an emergency admission were emergency procedures. Diagnoses of metastatic cancer in the 6 months before and 1 month after CRC surgery (ICD-10: “C780-C784,” “C786,” “C787,” “C79”) were used to identify patients with stage IV disease. The Royal College of Surgeons Charlson score was used to identify comorbidities based on diagnostic information in the operative admission and admissions in the preceding 6 months.^24^ The area of residence, defined according to the Office for National Statistics with a typical population of about 1500 people, was used to capture socioeconomic deprivation according to the national Index of Multiple Deprivation Quintiles (IMDQ).^25^

### Mortality and Other Surgical Outcomes

Outcomes analyses were restricted to patients undergoing a major resection (ie, patients undergoing a stoma or stent procedure only were excluded). The main outcome was death in hospital within 30 days of CRC resection. Patients with a recorded critical care episode during their admission were considered to have been admitted to critical care. Length of stay (LOS) was the number of days between the date of operative procedure and discharge from hospital. Unplanned readmissions and reoperations within 30 days of a resection were identified in HES using methods described previously.^26^

### Identification of COVID-19 Status

Either of the ICD-10 codes for confirmed or suspected COVID-19 infection (“U071,” “U072”) within the operative admission were used to indicate COVID-19 infection. It was not possible to differentiate between hospital-acquired infections and patients admitted with preexisting COVID-19 infection.

### Identification of “Hot” and “Cold” Hospital Sites

Information on “hot” and “cold” hospital sites came from a mapping exercise performed by the National Bowel Cancer Audit in June 2020.^27^ Clinical leads in each NHS hospital were asked for the location of all NHS and independent-sector COVID-19 “cold” sites available to their hospital in mid-April 2020. “Hot” and “cold” site information was available for 146 of the 148 NHS hospitals that provide CRC surgery in England. A further six independent-sector “cold” site hospitals were identified in the data as providing CRC surgery only since the start of the pandemic. For the two NHS hospitals that did not provide information on “hot” and “cold” sites, we relied on the information in the data on independent sector “cold” sites. This identified one of these hospitals as having access to a “cold” site.

### Statistical Analysis

Chi-squared tests were used to compare mortality after major resection before or during the pandemic. Multivariable logistic regression was used to model, separately for elective and emergency resections, the difference in surgical mortality before and during the COVID-19 pandemic, adjusted for case-mix factors (age, sex, stage IV disease, number of comorbidities, and socioeconomic deprivation), using robust standard errors to deal with clustering of data within hospitals. We assessed interactions between period of treatment (before or during the pandemic) and type of site (“hot” and “cold” site) using Wald tests. Complete case analyses were used because only 2% of patients undergoing CRC resection were missing data on any of the case-mix factors.

A sensitivity analysis was carried out in which a random intercept for hospital was included in each adjusted logistic regression model to reflect a variation in outcomes between hospitals. The adjusted results were little changed.

## RESULTS

Eleven thousand seven hundred three patients with CRC underwent surgery in the English National Health Service immediately before the COVID-19 pandemic and 3227 during the early COVID-19 pandemic period.

**FIGURE 1.**
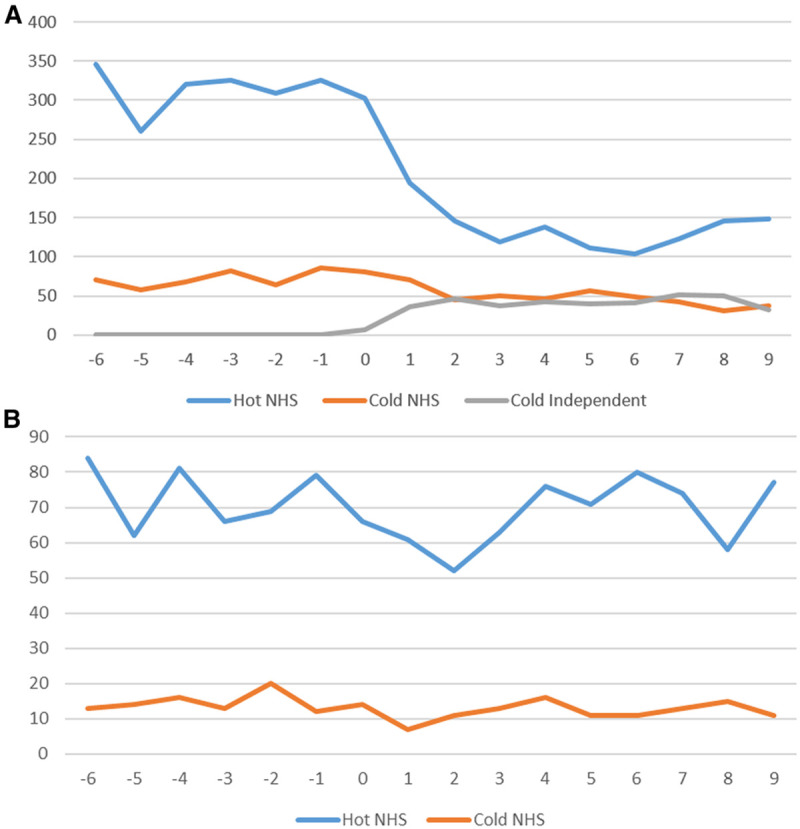
Number of CRC procedures performed per week in relation to the start of the COVID-19 pandemic (March 23, 2020: week 0) by “hot” and “cold” sites. A, Elective procedures and (B) emergency procedures. CRC indicates colorectal cancer; NHS, National Health Service.

The number of patients who underwent elective CRC surgery dropped sharply at the end of March 2020, decreasing by almost a half over a period of 3 weeks from on average 386 procedures a week in the 6 weeks before the pandemic to 214 procedures a week from week 3 of the pandemic (Figure 1). The number of emergency procedures dropped slightly at the end of March 2020 but returned to similar levels as before the pandemic within around 3 weeks from on average 88 procedures a week in the 6 weeks before the pandemic to 84 procedures a week from week 3 of the pandemic.

The reduction in elective procedures was predominantly in “hot” sites (Figure 1). Some independent sector hospitals started to function as “cold” sites for elective services from the end of March 2020 and contributed almost 50% of the “cold” site provision from mid-April 2020. Approximately one-sixth of all elective CRC surgery was provided in an independent sector “cold” site during the early pandemic period.

### Patient and Surgical Characteristics Before and During the COVID-19 Pandemic

There were only small differences in the characteristics of patients having elective surgery during the COVID-19 pandemic compared to before (Table 1). During the pandemic, these patients were on average slightly younger and had slightly fewer comorbidities. There was a small shift from elective anterior resections (from 31.0% before to 27.0% during the pandemic period) toward Hartmann’s procedures (from 5.8% to 11.1%). There was a substantial drop in the proportion of elective resections carried out laparoscopically (from 62.5% to 35.9%) and a small decrease in the proportion of patients who were recorded as admitted to critical care (from 7.8% to 5.1%).

**TABLE 1.** Characteristics of 14,930 CRC Patients Undergoing Surgery Immediately Before and During the COVID-19 Pandemic Period in the English National Health Service

		Elective Surgery				Emergency Surgery			
		Before Pandemic		During Pandemic		Before Pandemic		During Pandemic	
		N	%	N	%	N	%	N	%
**Total**		**9424**		**2427**		**2279**		**800**	
**Age group (y**)	**<50**	611	6.5	176	7.6	242	10.6	78	9.8
	**50–59**	1315	14.0	345	14.9	326	14.3	115	14.4
	**60–74**	4487	47.7	1110	47.9	890	39.1	305	38.2
	**75–84**	2446	26.0	594	25.7	590	25.9	215	26.9
	**≥85**	542	5.8	90	3.9	228	10.0	86	10.8
	**Missing**	23 (0.2)		112 (4.6)		3 (0.1)		1 (0.1)	
**Gender**	**Male**	5327	56.5	1358	56.0	1259	55.2	435	54.4
	**Female**	4097	43.5	1069	44.0	1020	44.8	365	45.6
**Tumor site**	**Caucus/appendix/ascending colon**	2563	27.2	671	27.6	623	27.3	221	27.6
	**Hepatic flexure**	404	4.3	102	4.2	108	4.7	33	4.1
	**Transverse colon**	607	6.4	168	6.9	191	8.4	75	9.4
	**Splenic flexure/descending colon**	447	4.7	101	4.2	232	10.2	87	10.9
	**Sigmoid colon**	1907	20.2	450	18.5	576	25.3	202	25.2
	**Rectosigmoid**	684	7.3	192	7.9	166	7.3	61	7.6
	**Rectum**	2731	29.0	711	29.3	326	14.3	98	12.3
	**Not classified**	81	0.9	32	1.3	57	2.5	23	2.9
**Stage IV CRC**		743	7.9	182	7.5	682	29.9	219	27.4
**Comorbidities**	**0**	4973	52.8	1348	55.5	1132	49.7	400	50.0
	**1**	2861	30.4	739	30.4	727	31.9	245	30.6
	**≥2**	1590	16.9	340	14.0	420	18.4	155	19.4
**Surgical procedure**	**Right hemi/transverse colectomy**	3572	37.9	940	38.7	842	36.9	304	38.0
	**Left hemicolectomy**	367	3.9	77	3.2	53	2.3	10	1.3
	**Sigmoid colectomy**	363	3.9	75	3.1	34	1.5	7	0.9
	**Total/subtotal colectomy**	299	3.2	81	3.3	110	4.8	37	4.6
	**Anterior resection**	2920	31.0	656	27.0	78	3.4	20	2.5
	**APER** **/pelvic exenteration**	711	7.5	168	6.9	15	0.7	4	0.5
	**Hartmann’s**	542	5.8	270	11.1	361	15.8	144	18.0
	**Stoma**	546	5.8	131	5.4	588	25.8	182	22.8
	**Stent**	104	1.1	29	1.2	198	8.7	92	11.5
**National quintiles socioeconomic deprivation (IMDQ**)	**1 (most deprived**)	1419	15.2	302	13.1	402	17.8	117	14.8
	**2**	1673	18.0	411	17.9	404	17.9	143	18.1
	**3**	1886	20.2	475	20.7	488	21.6	182	23.0
	**4**	2177	23.4	581	25.3	456	20.2	184	23.2
	**5 (most affluent**)	2165	23.2	531	23.1	506	22.4	166	21.0
	**Missing**	104 (1.1)		127 (5.2)		253 (1.0)		8 (1.0)	
**COVID-19 ICD-10 code recorded during operative admission**		36	0.4	47	1.9	11	0.5	50	6.3
**Total patients undergoing a major resection**		8774		2267		1493		526	
**Laparoscopic surgery performed**		5485	62.5	814	35.9	264	17.7	51	9.7
**Critical care admission**		687	7.8	116	5.1	192	12.9	60	11.4

APER, abdominoperineal excision of rectum; CRC, colorectal cancer; ICD-10, International Classification of Diseases, 10th revision; IMDQ, index of multiple deprivation quintiles.

There was relatively little change in the characteristics of patients undergoing emergency procedures during the pandemic (Table 1). There was a decrease in the proportion of resections carried out laparoscopically (from 17.7% to 9.7%), and a slight decrease in the proportion of patients recorded as admitted to critical care (from 12.9% to 11.4%).

### Mortality After Major CRC Resection

Table 2 shows that the overall 30-day inpatient mortality after an elective resection was slightly increased during the COVID-19 pandemic period (1.2%; 95% CI: 0.8% to 1.8%) compared with before (0.9%; 95% CI: 0.7% to 1.2%). After adjustment for age, sex, stage IV cancer, comorbidities, and socioeconomic deprivation, there was no statistical evidence of increased elective mortality (adjusted OR 1.54 [95% CI: 0.98 to 2.43], *P* = 0.063). Small, nonsignificant increases in 30-day inpatient mortality were seen both in “hot” sites (from 1.0% to 1.4%) and in “cold” sites (from 0.7% to 0.9%), and there was no statistical evidence of a difference in these increases between “hot” and “cold” sites (*P* for interaction 0.855).

**TABLE 2.** 30-day In-patient Mortality After Major CRC Resection Immediately Before and During the COVID-19 Pandemic Period

**Elective Surgery**											
	**Before Pandemic**			**During Pandemic**			**During Versus Before Pandemic**N = 10,823 patients with complete data on age, sex, stage IV cancer, comorbidities, and socioeconomic deprivation				
	**N**	**%**	**95% CI**	**N**	**%**	**95% CI**	**Crude Odds Ratio**	**Adjusted Odds Ratio**	**95% CI**	***P***	***P* Interaction**
All sites	8774	0.9	0.7–1.2	2267	1.2	0.8–1.8	1.35	1.54	0.98–2.43	0.063	
Hot sites	7221	1.0	0.8–1.2	1411	1.4	0.9–2.2	1.47	1.65	0.97–2.82	0.067	0.855
Cold sites	1553	0.7	0.4–1.3	856	0.9	0.4–1.8	1.29	1.49	0.57–3.88	0.413	
**Emergency Surgery**											
	**Before Pandemic**			**During Pandemic**			**During Versus Before Pandemic** **N = 1994 patients with complete data on age, sex, stage IV cancer, comorbidities, and socioeconomic deprivation**				
	**N**	**%**	**95% CI**	**N**	**%**	**95% CI**	**Crude Odds Ratio**	**Adjusted Odds Ratio**	**95% CI**	***P***	***P* Interaction**
All sites	1493	5.6	4.5–6.9	526	8.9	6.6–11.7	1.67	1.74	1.21–2.50	0.003	
Hot sites	1232	5.6	4.4–7.0	444	8.8	6.3–11.8	1.65	1.67	1.10–2.52	0.015	0.554
Cold sites	261	5.7	3.3–9.3	82	9.8	4.3–18.3	1.76	2.19	1.02–4.71	0.045	

CI, confidence intervals; CRC, colorectal cancer.

The overall 30-day inpatient mortality after emergency resection increased markedly during the pandemic (8.9%; 95% CI: 6.6% to 11.7%) compared with before (5.6%; 95% CI: 4.5% to 6.9%) and this difference remained statistically significant after risk adjustment (adjusted OR 1.74 [95% CI: 1.21 to 2.50], *P* = 0.003). The 30-day inpatient mortality increased both in “hot” sites (from 5.6% to 8.8%) and “cold” sites (from 5.7% to 9.8%), and again there was no statistical evidence of a difference in these increases between “hot” and “cold” sites (*P* for interaction 0.544).

### Other Surgical Outcomes

There were only minor, nonsignificant differences in unplanned readmissions and reoperations before and during the pandemic, regardless of urgency (Table 3; *P* always > 0.3). Hospital stays of over 14 days were much more frequent before the pandemic than during, particularly for elective patients (from 13.5% to 8.4%) but also for emergency patients (from 32.2% to 26.4%).

**TABLE 3.** 30-day Unplanned Readmission, Reoperation, and Length of Hospital Stay After Major CRC Resection Immediately Before and During the COVID-19 Pandemic Period

	**Before Pandemic**			**During Pandemic**			
	**Number**	**%**	**95% CI**	**Number**	**%**	**95% CI**	***P***
Elective Surgery							
Total	**8**774			**2**267			
30-d readmission	895	10.2	9.6–10.9	240	10.6	9.3–11.9	0.661
30-d reoperation	728	8.3	7.7–8.9	170	7.5	6.4–8.6	0.322
LOS >14 d	1188	13.5	12.8–14.3	191	8.4	7.3–9.6	<0.001
Emergency Surgery							
Total	**1**493			**5**26			
30-d readmission	163	10.9	9.4–12.6	62	11.8	9.2–14.9	0.572
30-d reoperation	165	11.1	9.6–12.8	60	11.4	8.8–14.4	0.858
LOS >14 d	481	32.2	29.9–34.7	139	26.4	22.7–30.4	0.014

CI, confidence intervals; CRC, colorectal cancer.

### Sensitivity Analysis

A random intercept for hospital was included in each adjusted logistic regression model to reflect a variation in outcomes between hospitals. The adjusted results were not materially changed.

### Patients With a Recorded COVID-19 Diagnosis

Overall, 144 patients had a confirmed or suspected COVID-19 diagnosis in their surgical record during the study period (Table 1). A small proportion had a recorded COVID-19 diagnosis before the pandemic period (0.4% of elective patients and 0.5% of emergency patients), and almost all of these had CRC surgery in March 2020. The proportion of patients with perioperative COVID-19 infection was considerably higher during the pandemic (1.9% elective and 6.3% emergency patients) and was higher in “hot” sites (2.5% elective and 6.6% emergency patients) than in “cold” sites (1.0% elective and 4.1% emergency patients) during the pandemic.

Of the patients with a confirmed or suspected COVID-19 diagnosis, 119 (82.6%) underwent major resection. Mortality in these patients was high, with one in seven patients who had elective surgery (13.9%; 95% CI: 7.2% to 23.5%) and one in four patients who had emergency surgery (25.0%; 95% CI: 12.7% to 41.2%) dying in hospital within 30 days (Table 4). Thirty-day reoperation rates and length of hospital stay were also considerably increased in these patients.

**TABLE 4.** 30-day In-patient Mortality, Unplanned Readmission, Reoperation, and Length of Hospital Stay After Major CRC Resection in 119 Patients With a Confirmed or Suspected COVID-19 Diagnosis*

**Elective Surgery**			
	**Number**	**%**	**95% CI**
Total	**79**		
30-d in-patient mortality	11	13.9	7.2–23.5
30-d reoperation	21	26.6	17.3–37.7
Median (IQR) length of stay (d)		17	10–32
**Elective Surgery**			
	**Number**	**%**	**95% CI**
Total	**40**		
30-d in-patient mortality	10	25.0	12.7–41.2
30-d reoperation	7	17.5	7.4–32.8
Median (IQR) length of stay (d)		20	11–32

*30-day readmission is not presented for patients with a confirmed or suspected COVID-19 diagnosis because such a high proportion died or were still in hospital within 30 days of surgery.

CI, confidence intervals; CRC, colorectal cancer; IQR, interquartile range.

## DISCUSSION

### Main Findings

In the first 2 months of the COVID-19 pandemic period in the English NHS, the number of patients undergoing elective CRC surgery halved, whereas the number undergoing emergency CRC surgery remained static. There was little change in the characteristics of patients treated during the pandemic, both for elective and emergency surgery, but there was a clear change in surgical practice with a sharp drop in the use of the laparoscopic approach and a shift from anterior resection with colorectal anastomosis for rectal cancer toward Hartmann’s procedure with colostomy.

The onset of the pandemic was also associated with an increase in mortality after major CRC resection, both in elective and emergency surgery. A key finding of our study is that this increase in surgical mortality did not appear to be mitigated by treating patients in designated “cold” sites at this stage of the pandemic. This is in contrast to an international cohort study that demonstrated that the provision of COVID-19 “cold” sites reduces the risk of hospital-acquired COVID-19 infection, pulmonary complications, and mortality.^28^

Mortality after CRC resection was very high in CRC patients who had a confirmed or suspected perioperative COVID-19 diagnosis with about one in seven dying after an elective and one in four after an emergency CRC resection.

### Limitations

The first limitation of this study was that detailed information on cancer stage, disease severity, or symptoms that may warrant earlier surgery, such as bleeding or obstruction, was not available. However, we did adjust for stage IV disease, age, sex, comorbidities, and socioeconomic deprivation. In line with other studies, we found little change in any of these characteristics during the pandemic.^16^ Prioritization for surgery of patients with more advanced disease and delayed presentation of emergency patients is likely to explain some of the increased mortality during the pandemic, but data were not available on nodal status or tumor size to be able to explore this.

Second, it is very likely that we under-report patients who had a COVID-19 diagnosis due to lack of available testing at the time and the high proportion of asymptomatic cases. Consequently, the main comparison of our study is between patients operated before and during the COVID-19 pandemic period rather than between patients with and without a COVID-19 diagnosis.

Third, there was no generally accepted definition of a “cold” site at the time of our study, which may have diluted the differences in outcomes between “hot” and “cold” sites. Further details on the characteristics of the “hot” and “cold” sites would have allowed a more robust comparison between them. “Cold” sites were being set up during the study period, and their defining characteristics may have developed over time. In most cases, they will eventually have included rapid access to testing for patients and hospital staff, separate clinical teams, geographical separation of COVID and non-COVID services and dedicated access to cold support services in patient transport, diagnostics, radiology, and critical care.^29^ Other preventative measures to protect patients against perioperative COVID-19, including preoperative COVID-19 testing, and presurgery household isolation may not have always been fully implemented, although they were advocated during the study period. All patients included in the study were NHS patients, whether they were treated in “hot” or “cold” sites, and other than the type of site for their surgical treatment, there is no reason to expect differences in their cancer care.

Fourth, it is likely that critical care admissions were under-recorded in HES data. Previous studies have estimated that around a third of patients go to critical care during their CRC surgery admission, whereas we estimated this to be around 10%.^30^ It is unlikely, however, that the under-reporting of critical care admissions would differ before and during the pandemic and our study can therefore provide an estimate of the relative reduction in access.

Fifth, information on cause of death was unavailable. This may have helped to understand the reasons for the increase in mortality during the pandemic.

Finally, in this study, the start of the pandemic was defined as March 23, 2020, the date of the UK “lockdown” period. As demonstrated in the study, this also coincides with the start of severe disruption to CRC services in the United Kingdom. However, it is known that the first cases of COVID-19 in the populations were before this.^31,32^ It was not possible to carry out additional analyses to explore the effect of changing this start date because shifting the date earlier would make the number of deaths in cold sites before the pandemic too small to be able to make reliable comparisons.

### Findings on the Disruption of CRC Surgery

Limited access to elective surgery has been widely recognized during the COVID-19 pandemic with one study reporting that about one-third of CRC surgeries were canceled or postponed in the 12 weeks of peak disruption.^1^ Our national data showed an even greater reduction in the overall numbers of patients undergoing CRC surgery during the first months of the pandemic. However, in line with other studies, we found that the number of patients undergoing emergency treatment was hardly affected.^33–35^

Our study indicates that there was sufficient capacity, in terms of access to hospital beds, emergency theater provision, and perioperative care either in “hot” or “cold” sites, to ensure that most patients with an emergency presentation of CRC were relatively protected and could proceed to surgery without significant delay, which is especially important for the management of patients with an acute condition. However, capacity for elective CRC surgery halved, which was driven predominantly by the diversion of healthcare resources and staff toward the management of COVID-19 patients and measures put in place to prevent COVID-19 transmission. It is unlikely that this diversion from elective care will be as prominent in future resurgences of the pandemic, given that in many countries COVID-19 vaccination of vulnerable groups will reduce the pressure on hospital capacity.^36^ Recent modeling also suggests that preoperative vaccination of all elective surgical patients may greatly reduce the risks of hospital-acquired postoperative COVID-19 infection.^37^

Initial national guidelines issued for the United Kingdom indicated that all non-emergency CRC surgery (ie, treatment not required within 72 hours) may be delayed by up to 12 weeks without major prognostic implications, and this guidance may have further contributed to these reductions.^38^ However, subsequent evidence has suggested that a 12-week delay might reduce overall survival for CRC patients by up to 19%.^39^

Temporizing treatment strategies, such as short-course radiotherapy with prolonged wait to surgery, were used to minimize the effect of delaying CRC resection.^17^ Patients with locally advanced rectal cancer who might previously have been considered for pelvic exenteration were deferred because of the resource intensity of this procedure and the expectation that outcomes would be negatively affected in the event of acquiring nosocomial COVID-19 infection during a more prolonged hospital stay. Similarly, patients with metastatic disease may have been offered stenting and systemic anticancer therapy or best supportive care rather than palliative resection of a symptomatic primary tumor.^40, 41^

In addition to a reduction in the number of surgical procedures, there was a substantial decrease in laparoscopic access and an increase in stoma rates. These changes followed initial national professional guidance advocating against laparoscopy and in favor of risk-averse surgery to reduce critical care admissions for anastomotic complications.^42^ Recent evidence suggests that adequate personal protective equipment, and closed-loop insufflation systems or filtration devices, may facilitate safe laparoscopic procedures during the COVID-19 pandemic. Laparoscopic surgery is therefore unlikely to be reduced during later waves of COVID-19, given its advantages in reduced length of stay and lower risk of pulmonary sequela.^15, 43^

### Findings on the Effectiveness of Measures to Mitigate COVID-19 Risk

Our study found that despite measures to mitigate COVID-19 risk in CRC patients undergoing surgery, such as the introduction of COVID-19 surgical “cold” sites, surgical mortality was higher during the first 2 months of the pandemic period, especially in patients who needed emergency treatment. This finding corresponds to earlier findings in much smaller studies in the United Kingdom and Spain.^33, 34^

This study did not find evidence of a difference in surgical mortality between patients treated in “hot” and “cold” sites. Further data will be needed to adequately power such comparisons. Other studies have demonstrated that the provision of COVID-19 “cold” sites reduces the risk of hospital-acquired COVID-19 infection and pulmonary complications.^15, 28, 44–46^

It is likely that the increase in mortality after CRC resections during the COVID-19 pandemic is related to several factors. First, our results confirm that COVID-19 infection greatly increased surgical mortality.^11^ However, COVID-19 infection itself can only explain a very small part of the observed increases in mortality. Based on our findings, it can be estimated that approximately 20% of the patients who had emergency CRC resection needed to be infected to fully explain the observed increases in surgical mortality, whereas the population COVID-19 infection level was about 0.25% in England at the time.^47, 48^ This very small contribution of COVID-19 infection is also one of the most likely explanations of our observation that the increases in mortality after CRC resections during the COVID-19 pandemic period were very similar in “hot” and “cold” sites.

Second, we only found small decreases in patients moving to critical care after an elective CRC resection during the pandemic. Although other studies have found larger reductions in critical care use for patients undergoing elective procedures,^16^ our finding suggests that restricted access to critical care facilities can only be a minor explanation for the observed increases in CRC surgical mortality.

Third, a further possible explanation for the increases in surgical mortality during the pandemic is that operating with personal protection equipment caused visual, communication, and dexterity issues, which interfered with the quality of surgery.^49^ However, there was no increase in the reoperation rate either for elective or for emergency CRC resections that would also have been expected if wearing personal protection equipment had affected the safety of surgery. Similarly, redeployment of theater staff and lack of specialized theater teams accustomed to working together may have been a contributor to the human factor aspects, especially in emergency surgery. It is also possible that the shift in where patients were treated during the pandemic was toward hospitals with a different quality of care, although the majority of surgeons operating in the independent sector in the United Kingdom are NHS surgeons who carry out both NHS and private work.

Fourth, reluctance of patients to access healthcare during the pandemic may have led to late presentation and an increase in nutritionally and physiologically decompensated patients presenting for emergency surgery. And triage of elective patients based on clinical urgency is likely to have led to patients with more advanced disease undergoing resection during the pandemic. These factors may further explain some of the increased surgical mortality.

## CONCLUSIONS

Surgical resection is the mainstay of treatment for non-metastatic CRC. We found a halving of elective CRC procedures and a substantial increase in surgical mortality during the first 2 months of the COVID-19 pandemic. Both are of significant concern for CRC patients and clinical services, demonstrating the importance of maintaining CRC services and minimizing surgical risk during further resurgences of the pandemic. The likelihood of such resurgences depends on the effectiveness of vaccination strategies and the emergence of new COVID-19 variants.

The concept of using safe “cold” surgical sites, and the strategies needed to implement these, will need ongoing refinement to ensure implementation of maximum resilience for CRC surgery services during future waves of the COVID-19 pandemic.

The significantly worse outcomes during the pandemic cannot be explained directly by COVID-19 infection. Further study will be needed to understand the most likely factors responsible for this marked deterioration but may include the negative impact of personal protection equipment and constrained theater teams on surgeon performance, delayed clinical presentation and lack of safeguarding of the entire perioperative care pathway for high-risk CRC patients.

## ACKNOWLEDGMENTS

The National Bowel Cancer Audit is commissioned by the Healthcare Quality Improvement Partnership (HQIP) as part of the National Clinical Audit and Patient Outcomes Programme.
